# Big Genomes Facilitate the Comparative Identification of Regulatory Elements

**DOI:** 10.1371/journal.pone.0004688

**Published:** 2009-03-04

**Authors:** Brant K. Peterson, Emily E. Hare, Venky N. Iyer, Steven Storage, Laura Conner, Daniel R. Papaj, Rick Kurashima, Eric Jang, Michael B. Eisen

**Affiliations:** 1 Department of Molecular and Cell Biology, University of California, Berkeley, California, United States of America; 2 Genomics Division, Ernest Orlando Lawrence Berkeley National Laboratory, Berkeley, California, United States of America; 3 Department of Ecology and Evolutionary Biology, University of Arizona, Tucson, Arizona, United States of America; 4 Pacific Basin Agricultural Research Center, United States Department of Agriculture, Hilo, Hawaii, United States of America; 5 Howard Hughes Medical Institute, University of California, Berkeley, California, United States of America; 6 California Institute of Quantitative Biosciences, University of California, Berkeley, California, United States of America; 7 Center for Integrative Genomics, University of California, Berkeley, California, United States of America; Indiana University, United States of America

## Abstract

The identification of regulatory sequences in animal genomes remains a significant challenge. Comparative genomic methods that use patterns of evolutionary conservation to identify non-coding sequences with regulatory function have yielded many new vertebrate enhancers. However, these methods have not contributed significantly to the identification of regulatory sequences in sequenced invertebrate taxa. We demonstrate here that this differential success, which is often attributed to fundamental differences in the nature of vertebrate and invertebrate regulatory sequences, is instead primarily a product of the relatively small size of sequenced invertebrate genomes. We sequenced and compared loci involved in early embryonic patterning from four species of true fruit flies (family Tephritidae) that have genomes four to six times larger than those of *Drosophila melanogaster*. Unlike in *Drosophila*, where virtually all non-coding DNA is highly conserved, blocks of conserved non-coding sequence in tephritids are flanked by large stretches of poorly conserved sequence, similar to what is observed in vertebrate genomes. We tested the activities of nine conserved non-coding sequences flanking the *even-skipped* gene of the teprhitid *Ceratis capitata* in transgenic *D. melanogaster* embryos, six of which drove patterns that recapitulate those of known *D. melanogaster* enhancers. In contrast, none of the three non-conserved tephritid non-coding sequences that we tested drove expression in *D. melanogaster* embryos. Based on the landscape of non-coding conservation in tephritids, and our initial success in using conservation in tephritids to identify *D. melanogaster* regulatory sequences, we suggest that comparison of tephritid genomes may provide a systematic means to annotate the non-coding portion of the *D. melanogaster* genome. We also propose that large genomes be given more consideration in the selection of species for comparative genomics projects, to provide increased power to detect functional non-coding DNAs and to provide a less biased view of the evolution and function of animal genomes.

## Introduction

**Figure 1:**
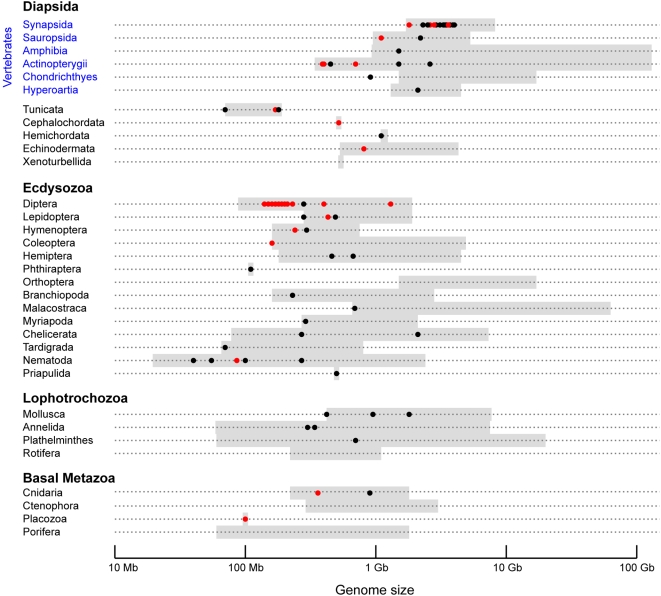
Animal genomes and sequenced animal genomes vary greatly in size. Genome size ranges for selected animal phyla (and other major taxonomic grouping) are shown as grey bars. Genome size data is from the Animal Genome Size Database [1]. Circles show sizes of genomes whose sequences have been published (red circles) or in progress (black circles). In progress genomes were obtained from National Human Genome Research Institute and the Department of Energy's Joint Genome Institute.

Animal genomes differ considerably in size, ranging from 20 million to over 100 billion basepairs [1], with significant variation between even closely related species (see Figure 1). This diversity is reflected in sequenced animal genomes, which currently range from the nematode *Meloidogyna incognita* at around 80 Mb to humans at around 3.2 Gb, with a marked difference in the sizes of sequenced genomes of invertebrates (most are smaller than 250 Mb) and vertebrates (most are larger than 1 Gb).

This taxa-specific bias in the sizes of sequenced genomes partially reflects taxa-specific differences in genome sizes. Few vertebrates, and no tetrapods, are known to have genomes smaller than 1 Gb, while most large invertebrate taxa contain species with far smaller genomes. It is still not clear why these differences exist, although several explanations have been proposed [2], [3]. However these broad trends in genome size do not fully account for the bias in the sizes of sequenced genomes.

The focus of early animal genome sequencing were the major model species. While the primary vertebrate species of interest – humans (3.0 Gb), mice (2.5 Gb), frogs (1.5 Gb) and zebrafish (1.5 Gb) – all have typically sized genomes for their taxa, the first two invertebrate species sequenced - *Drosophila melanogaster* (175 Mb) and *Caenorhabditis elegans* (100 Mb) have remarkably small genomes even when compared, respectively, to other flies and roundworms. Their small genomes are likely related to the features – rapid generation time, small body size and ease of genetic analysis – that make them ideal laboratory species [2]. Whatever the reasons, these differences in genome size fostered an impression that persists today that small genomes are a fundamental property of invertebrates.

This bias towards sequencing small invertebrate genomes persisted as sequencing moved beyond these initial candidates. The explanation is obvious - the cost of sequencing scales more or less linearly with the number of basepairs to be sequenced. Thus, where possible, genome sequencing projects have focused on species with small genomes – either by identifying species with small genomes within target taxa, or by ignoring taxa where no species with small genomes can be identified. While size has been a criterion in the selection of vertebrate species to sequence, it has been given far less weight in relation to the targeting of species of medical and agricultural import or value in annotating the human genome.

Several trends have emerged from the comparison of animal genomes of different size. Genome size is strongly correlated with repetitive DNA content [2], [3], [4], presumably because variation in genome size is driven by the expansion of repeat families or the purging of repetitive DNA [3], [5]. Where genomes differ in size, the scaling is not uniform: there is considerably more variation in the sizes of introns and intergenic DNA than in the amount of protein-coding DNA [2] (see Table S1 and Table S2). Smaller genomes thus have far less non-coding DNA and, assuming that the amount of functional non-coding DNA is relatively constant, a larger fraction of their non-coding DNA is functional. Genome-wide analyses of non-coding evolutionary constraint in different taxa support this interpretation: only 5 percent of the basepairs in human non-coding DNA appear to be under evolutionary constraint [6], while approximately 50 percent of the twenty-fold smaller *D. melanogaster* genome is similarly constrained [7].

These size-specific differences in genome organization have practical consequences for many aspects of the analysis and annotation of genome sequences. Analysis of non-coding DNA from the human, mouse and other vertebrate genomes suggests that a large fraction of evolutionary conserved regions therein are involved in transcriptional regulation, and there are now myriad examples of vertebrate enhancers identified through experimental analysis of conserved non-coding DNAs (e.g. [7], [8], [9], [10], [11], [12]). However, the published record – and our experience [13] - suggests that these methods are far less effective in invertebrates. Significant resources have been devoted to sequencing species related to *D. melanogaster* and *C. elegans* [14], [15]. While there have been some successes in these organisms (e.g. [16]), comparative genomic methods have not yielded the expected bounty of regulatory sequences.

**Figure 2:**
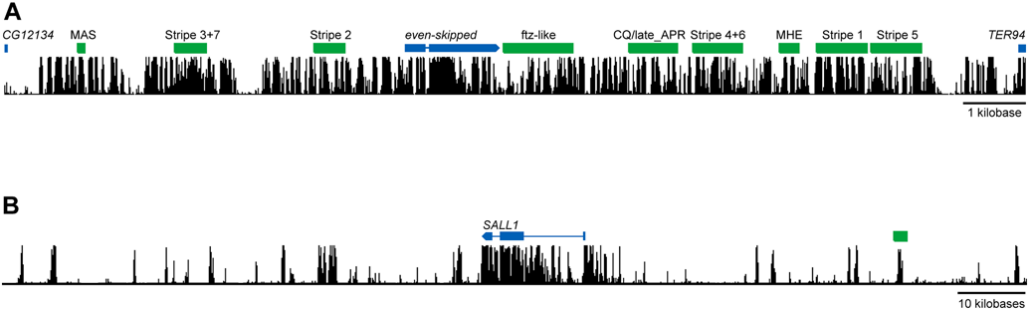
Landscape of sequence conservation in vertebrates and *Drosophila*. Posterior probabilities of selective constraint are plotted across illustrative loci in *Drosophila* and vertebrates (computed with PHASTCONS [44]; data obtained from UCSC genome browser). Blue annotations indicate coding regions, green indicate experimentally validated enhancers. A) Genomic interval surrounding the *D. melanogaster even-skipped* gene (conservation shown is for 12 *Drosophila* species plus *Anopheles*, *Apis*, and *Tribolium*). Several confirmed *eve* enhancers are shown, drawn from the RedFly database [28], [47]. B) Approximately 150 kb of the human *SALL1* locus (conservation shown is across all vertebrates). The midbrain and neural tube enhancer depicted here is from [48].

A comparison of the landscape of non-coding conservation in the human and *D. melanogaster* genomes (Figure 2) suggests an explanation for the differential effectiveness of comparative regulatory sequence identification in these two species. Human non-coding DNA generally consists of small stretches of conservation separated by relatively large swaths of non-constrained DNA. It is thus easy to identify conserved non-coding sequences that are candidates for experimental analysis. In contrast, non-coding DNA in the *D. melanogaster* genome is far more uniformly conserved. This both suggests that nearly all *D. melanogaster* non-coding DNA is functional, and obscures the boundaries between functional elements that could be used to identify candidate regulatory sequences.

As part of a project to study the evolution of transcriptional enhancers, we sequenced the orthologs of several genes involved in early pattern formation in *D. melanogaster* in other families of flies [17]. Our selection of species for this project was guided by phylogenetic position, availability of material for sequencing, and the suitability of the species for subsequent experimental analysis. Incidentally, species in one of the families we targeted - the Tephritidae, or “true fruit flies” - turned out to have relatively large genomes - four to six times larger than *D. melanogaster*. As this family, which diverged from the *Drosophila* lineage approximately 150 million years ago, contains many agricultural parasites of significant economic import, such as the Mediterranean fruit fly *Ceratitis capitata*, abundant material was available for sequencing and follow-up experiments. This practical advantage offset the investment of time and resources required to work with their relatively large genomes.

**Figure 3:**
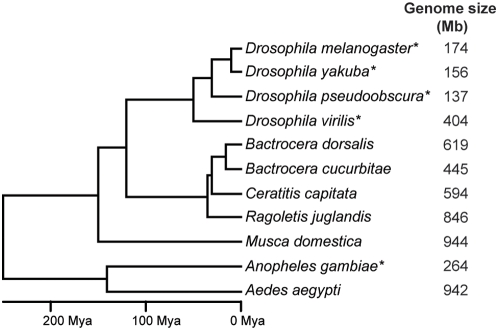
Tephritid genomes are larger than *Drosophila* genomes. Phylogenetic relationships and approximate divergence times of several dipteran species (left) are shown along with experimentally determined haploid genome sizes (right), drawn from the Animal Genome Size Database [1] (*Drosophila spp*, *M. domestica*, *A. aegypti*, *A. gambiae*), and our own experiments (*Bactrocera spp*, *C. capitata*, *R. juglandis*). While some groups (*Drosophila*, *Anopheles*) have undergone substantial reduction in genome size, many closely related species including the tephritids described here have substantially larger genomes. Asterisks indicate species with available whole-genome sequence.

The four tephritid species we selected (Figure 3) span an evolutionary distance roughly comparable to that spanned by sequenced *Drosophila* species. Thus when we examined the first set of tephritid sequences, we were surprised to see that tephritids did not have the nearly-continuous non-coding sequence conservation observed in *Drosophila*. They appeared instead to have the same islands of non-coding sequence conservation flanked by large regions of rapidly evolving DNA observed in humans.

In this paper we explore this observation, its consequences for the identification of regulatory sequences in animal genomes, and the implications for species selection in comparative genomics projects.

## Results

### Tephritid Genomes are Substantially Bigger than *D. melanogaster*

We obtained adult samples of four tephritid species from laboratory stocks (*Ceratitis capitata*, *Bactrocera dorsalis*, *Bactrocera cucurbitae*) or field collection (*Ragoletis juglandis*). We used propidium-iodide staining and flow cytometry to determine the sizes of the genomes of each species, which ranged from 440 to 850 Mb (Figure 3). We then generated fosmid libraries for each species, and screened for 20 genes involved in anterior-posterior segmentation, heart specification and extraembryonic tissue formation. Of these, we recovered four genes from three or more tephritid species: orthologs of the *D. melanogaster even-skipped* (*eve*), *giant* (*gt*), *pannier* (*pnr*) and *Dorsocross* (*Doc1*) genes (Table 1).

**Table 1:** Loci sequenced for this study.

Gene	Species	# fosmids	Size of sequenced region
*eve*	*C. capitata*	2	57436
	*B. dorsalis*	1	33916
	*B. cucurbitae*	2	64056
	*R. juglandis*	2	60091
*gt*	*B. dorsalis*	1	39815
	*B. cucurbitae*	1	36762
	*R. juglandis*	2	33234
*pnr*	*C. capitata*	1	41535
	*B. dorsalis*	3	62446
	*B. cucurbitae*	1	39678
	*R. juglandis*	1	38746
*doc*	*C. capitata*	1	30000
	*B. cucurbitae*	1	32750
	*R. juglandis*	2	44379

While the sizes of *D. melanogaster* loci in this set (distance from next upstream to next downstream gene) ranged from 11 to 26 kb, we frequently required two or more 40 kb fosmids to span entire tephritid loci. For example, the *D. melanogaster eve* locus is 11 kb, while the *C. capitata eve* locus is 48 kb (Table 2). The difference in locus size is roughly proportional to the difference in genome size, and the larger size of tephritid loci is primarily due to increases in the size of introns and intergenic regions, and not of coding DNA (Table 2).

**Table 2:** Sizes of *even-skipped* locus (MAS enhancer to stripe 4/6 enhancer).

Species	Locus (kb)	Upstream (kb)	Downstream (kb)	Coding (aa)
*D. melanogaster*	11,146	5,918	3,078	377
*D. erecta*	11,129	5,880	3,110	379
*D. ananassae*	9,932	4,840	2,959	382
*D. pseudoobscura*	11,391	5,306	3,624	360
*D. virilis*	14,041	6,677	5,128	366
*C. capitata*	48,191	19,785	22,244	342

**Table 3:** Conserved non-coding sequenced in *C. capitata even-skipped* locus

Scaffold (Genbank Accession)	Start	End	Size	Name
FJ710597	4422	6807	2386	eve-1
FJ710597	9961	12014	2054	eve-2
FJ710597	15766	17854	2089	eve-3
FJ710597	23489	25772	2284	eve-promoter
FJ710597	39976	41925	1950	eve-4
FJ710597	43936	44699	764	eve-5
FJ710597	45427	46590	1164	eve-6
FJ710597	48794	49949	1156	eve-7
FJ710597	50896	52270	1375	eve-8
FJ710597	52567	54685	2119	eve-9

### Landscape of Non-coding Conservation in Tephritids

**Figure 4:**
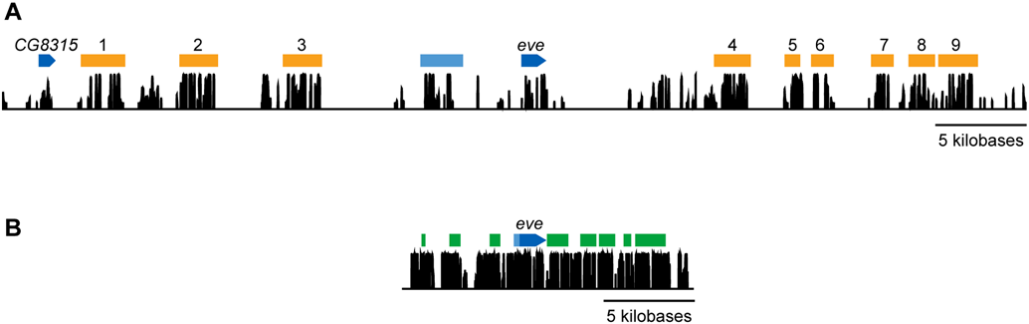
Landscape of sequence conservation in tephritids and *Drosophila* (eve). A) Phastcons [44] (version v0.9.9.6b) estimated posterior probabilities of conservation in four tephritids for 60 kb surrounding the *C. capitata eve* gene. Blue annotations indicate coding regions, conserved intervals are shown in orange. The interval numbers are used throughout the text. The presumptive *C. capitata* basal promoter is shown in light blue. B) *D. melanogaster eve* locus conservation plot computed with phastCons (rho 0.25) [44], rendered to scale with *C. capitata* plot in panel A, showing comparable highly conserved content but with virtually all intervening non-conserved DNA absent in *Drosophila*. Redfly enhancers listed in Figure 2 are shown in green and the basal promoter in light blue.

The pattern of conservation across each of the tephritid loci is markedly different than those of their *D. melanogaster* orthologs (see Figure 4 and Figure S1). In each case, there are numerous highly conserved intervals of one to two kb in size separated by equal or greater lengths of non-conserved DNA, as is observed in vertebrates (Figure 2).

To quantify differences in non-coding constraint in *Drosophila*, tephritids and vertebrates, we identified conserved non-coding regions of the human, *D. melanogaster* and *C. capitata* genomes using identical methods and sets of comparison species at roughly comparable distances. We then compared the distribution of the sizes of conserved blocks and the spacing between them in the three taxa.

**Figure 5:**
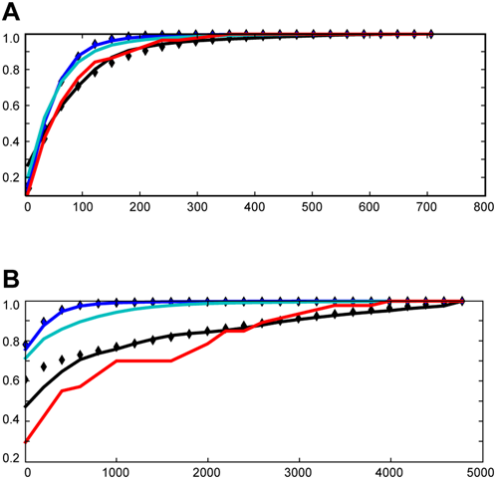
Size and spacing of highly conserved regions of human, *Drosophila* and tephritid genomes demonstrate global differences in constraint landscapes. Cumulative sums of normalized histograms are displayed for the sizes of conserved blocks (panel A) and the distances between them, i.e. the sizes of non-conserved intervals (panel B). Distributions of conserved region sizes are similar for *Drosophila*, *C. capitata* and human. Spacing between conserved regions, however, shows very different distributions in *Drosophila* and human; *C. capitata* conserved element spacings are similar to those observed in the human genome. Distributions are shown for UCSC phastCons “most conserved” tracks for human (black diamonds) and *D. melanogaster* (blue diamonds) as well as for phastCons run in-house on tephritid alignments (red line). In addition, *D. virilis* conserved block sizes and spacing (cyan line in panels A and B) are shown in order to assess the utility of a species with a large genome in supplying inter-element spacing information akin to vertebrates and tephritids (see text). In-house alignments and phastCons data are similarly displayed for *D. melanogaster* referenced *Drosophila* alignments (blue line) and for human referenced vertebrate alignments in 1% of the human genome (black line) in order to establish consistency between our analyses and UCSC datasets.

The size of conserved blocks is similar in these three species (Figure 5A). However, the spacing between conserved blocks is substantially smaller in *D. melanogaster* than in humans or *C. capitata* (Figure 5B), confirming our initial impression that the landscape of non-coding conservation is tephritids is more similar to typical vertebrates than to *Drosophila*.

### Native Expression of Developmental Genes in Tephritids

Of the genes for which we had data from multiple tephritid species, the regulation of *eve* is particularly well understood. Before evaluating the regulatory function of conserved blocks in the tephritid *eve* locus, we examined the endogenous expression patterns of *eve* in *C. capitata* embryos. We obtained embryos of *C. capitata* from large captive populations maintained for sterile-release programs, and modified *D. melanogaster* protocols for collection, fixation and whole-mount mRNA *in situ* hybridization to accommodate the roughly 5-fold greater size of tephritid embryos.

**Figure 6:**
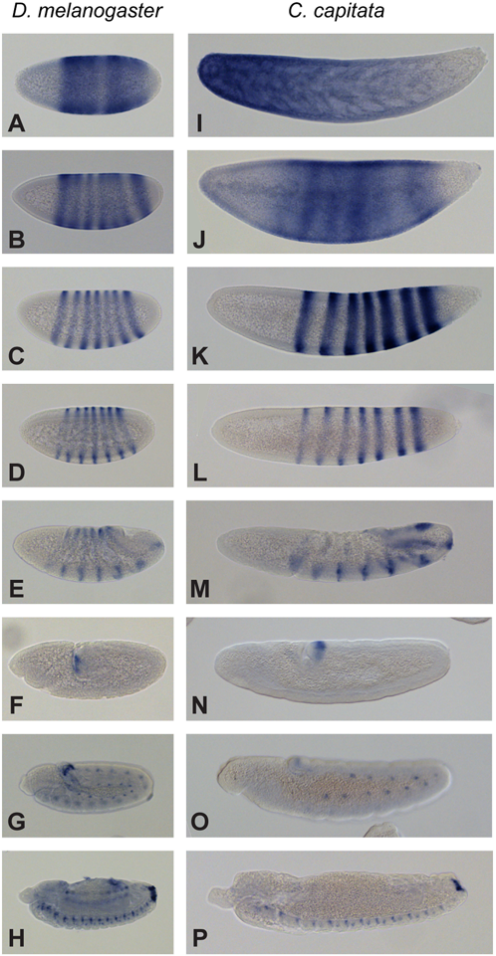
Native expression pattern of *eve* in *Drosophila melanogaster* and *Ceratitis capitata*. *even-skipped* expression patterns in *D. melanogaster* (A–H) and *C. capitata* (I–P) embryos were visualized by *in situ* hybridization with species-specific digoxigenin-labeled antisense RNA probes. While clear differences are manifest in the extremely early phases of expression (*D. melanogaster* stage 4–5, fixed 2–4 h AEL panels A,B; *C. capitata* fixed 0–8 h AEL panels I, J), Previously characterized epochs of *eve* expression appear substantially conserved. Parasegmental expression is conserved in the blastoderm and gastrulating embryo (*D. melanogaster* fixed 0–4 h AEL panels C, D and E, respectively, *C. capitata* fixed 8–32 h AEL panels K, L and M, respectively). So too is the post-gastrula expression domain of *eve* in the posterior, and in mesodermal lineages of the germ band extended embryo (*D. melanogaster* fixed 0–18 h AEL panels F, G, *C. capitata* fixed 8–32 h AEL panels N, O) and the neuronal and anal plate ring expression domains in the late embryo (*D. melanogaster* fixed 0–18 h AEL panel H, *C. capitata* fixed 26–50 h AEL panel P).

The native expression of *eve* in *C. capitata* embryos is shown in Figure 6. After accounting for differences in embryo geometry, there is broad conservation of *eve* expression between tephritids and *Drosophila* from the early establishment of pair-rule stripes and subsequent stripe refinement throughout the late embryonic domains of *eve* expression (neuronal, pericardial and anal plate).

The ease with which embryos could be collected for such studies is worth noting: a single gravid female would lay hundreds of eggs in a single morning on a moist sponge, meaning many grams of coarsely staged embryos can be gathered from population cages in one day.

### Tephritid Conserved Non-Coding Sequences Function as Enhancers in Transgenic *D. melanogaster* Embryos

We identified nine conserved non-coding sequences in the *C. capitata eve* locus (Figure 4; Table 3). Although ideally we would have evaluated the activity of these CNSs in transgenic tephritid embryos, robust methods for such analyses were not available. However, given the conservation of *eve* expression between *D. melanogaster* and *C. capitata*, and the success we and others [17], [18], [19], [20] have had in assaying the function of non-*Drosophila* dipteran enhancers in transgenic *D. melanogaster* embryos, we generated transgenic *D. melanogaster* lines for each tephritid *eve* CNS. In each construct a CNS was attached to the *D. melanogaster eve* promoter and a reporter gene.

**Figure 7:**
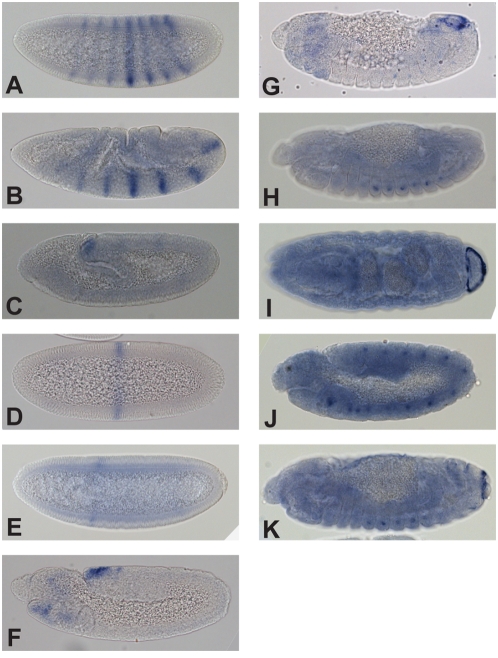
Expression patterns driven by tested *eve* fragments. Expression of reporter transcript in transgenic *D. melanogaster* embryos expressing either CFP or lacZ under the control of *C. capitata* conserved fragments and the naïve *D. melanogaster eve* basal promoter were visualized by *in situ* hybridization with digoxigenin-labeled antisense RNA probes. We tested all 9 fragments labeled in Figure 4. A–C) CFP expression driven by conserved fragment 1 (see Figure 4A) in blastoderm, gastrulating and germ-band extended embryos is entirely consistent with that of the *D. melanogaster* Minimal Autoregulatory Sequence (MAS; see Figure 2 in [24]). D) Conserved fragment 2 drives LacZ expression in the domain of the *eve* third parasegmental stripe, reminiscent of the activity of the *D. melanogaster* stripe 3+7 element (see Figure 2 in [22]), although the seventh stripe is not observed. E) CFP driven by conserved fragment 3 recapitulates the expression of the second stripe, along with weaker and incompletely penetrant expression in the domain of the seventh stripe, consistent with that driven by the *D. melanogaster* stripe 2 element (MSE; see Figure 2 in [21]). F,G) Conserved fragment 6 drives lacZ expression in the early anal plate ring as observed in the *D. melanogaster* eAPR enhancer (H–K). Segmental neuronal (H,J) and late anal plate ring (APR, I,K) CFP expression is observed in fragments 7 (H,I) and 8 (J,K). Fragment 7 neuronal expression (H) appears after germ-band retraction, and is primarily localized to EL neurons, while fragment 8 neuronal expression (J) appears earlier, and in both EL and CQ neurons. These activities are consistent with *D. melanogaster* EL neuronal and CQ neuronal/late APR enhancers (see Figure 3 in [23]). Fragment 4 drives fat body expression (data not shown); *eve* is not expressed in the fat body in *D. melanogaster* or *C. capitata*. Interestingly, the *ftz*-like element in *D. melanogaster* is also located in this region between the end of the coding sequence and the next annotated enhancer. The *ftz*-like element also drives expression that does not overlap with native *eve* expression. It should be noted that the fat body from *C. capitata* does not map to the *ftz* element. Fragments 5 and 9 drove no expression. Fragment 9 maps to the proximal half of the stripe 4+6 enhancer. We were missing comparative data beyond this fragment so it is possible that this conserved region extends distally and that we cloned an incomplete enhancer.

Seven of the nine tested *C. capitata eve* fragments drive patterns in *D. melanogaster* embryos (Figure 7). Six drive patterns that correspond to known *D. melanogaster* enhancers: the stripe 2 [21], stripe 3 [22], eAPR, EL neuronal and CQ neuronal/late APR enhancers [23], as well as the minimal autoregulatory sequence (MAS) [24]. One fragment drives expression in the fat body. As *eve* is not expressed in the fat body in *D. melanogaster* or *C. capitata*, this is probably not a *bona fide* enhancer.

As a control for the specificity of comparative enhancer identification in tephritids, we examined the activity of three non-conserved fragments. None of these fragments drove expression in *D. melanogaster* embryos.

### Mapping Tephritid Regulatory Sequences to the *D. melanogaster* Genome

Given the effectiveness of tephritid sequence comparisons in identifying enhancers, and the clear functional homology of many tephritid and *Drosophila* enhancers, we were interested in whether comparative genomics in tephritids might be used to annotate *Drosophila* non-coding DNA.

**Figure 8:**
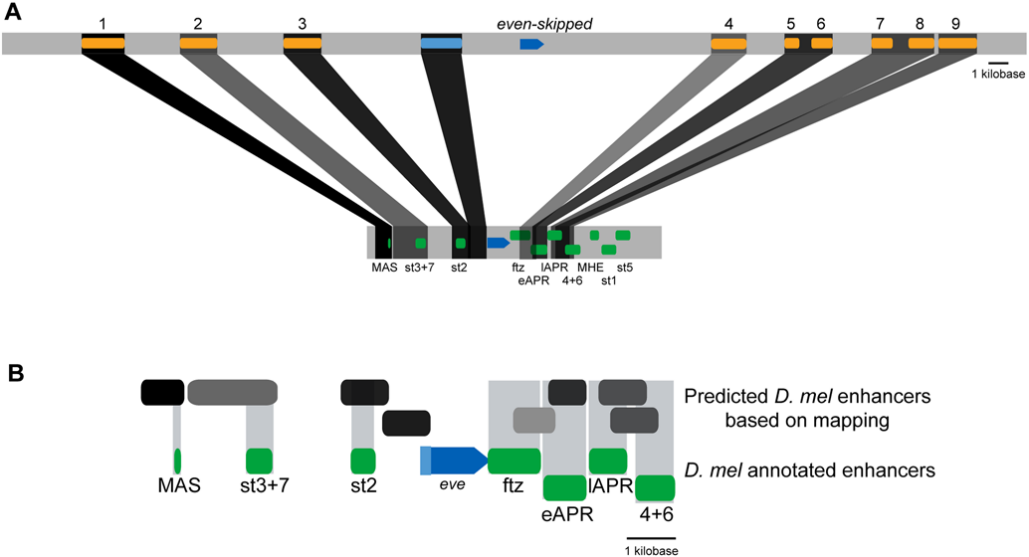
Mapping tephritid *eve* enhancers to *D. melanogaster*. A) Aggregate scoring of short, non-significant BLAST HSP and unique K-mer matches between the *C. capitata* (top) and *D. melanogaster* eve loci (bottom) was employed as described above to generate an orthology mapping of non-coding regions flanking the *eve* gene. Dark grey bars with opacity proportional to relative confidence of mapping link the best match regions between families. Orange annotations in *C. capitata*, top, indicate the cloned conserved fragments (numbering as employed throughout this work). Green annotations, in *D. melanogaster*, bottom, are confirmed enhancers drawn from the RedFly database [28], [47] (MAS: eve_mas; st3+7: eve_stripe_3+7; st2: eve_stripe2; ftz: eve_ftz-like; eAPR: eve_early_APR; CQ: eve_CQ/late_APR; 4+6: eve_stripe_4+6; MHE: eve_MHE; st1: eve_stripe1; st5: eve_stripe5). B) Zoom in on *D. melanogaster* locus showing mapped tephritids CNSs (grey, shading reflects mapping score) and known *D. melanogaster* enhancers [28] (green).

To do this, it would be necessary to map conserved non-coding sequences from tephritids to their orthologous regions in the *D. melanogaster* genome. Unfortunately, *Drosophila* and tephritid genomes are significantly diverged, such that primary comparison methods like BLAST do not discover significant non-coding similarity between the *D. melanogaster eve* locus and any tephritid species . However, we found that there were numerous short stretches of sequence similarity that, when considered in aggregate, reliably matched each new tephritid *eve* enhancer to a single region in the *D. melanogaster eve* locus (Figure 8; similar maps for the other factors are shown in Figure S1). Strikingly, the expression patterns of the tephritid enhancers corresponded to the expression patterns driven by *D. melanogaster* enhancers in the regions to which they were mapped (Figure 8).

## Discussion

### The Value of Big Genomes in Comparative Genomics

When we began working with tephritid genomes, we viewed their large size as an annoyance that necessitated the screening of an unusually large (compared to *Drosophila*) number of clones to identify genes of interest. However, once we began exploring the landscape of non-coding conservation between tephritid species to that between *Drosophila* species, we realized that large genomes provide several advantages for the comparative annotation of non-coding DNA.

Identifying regulatory sequences in large and small genomes represent two fundamentally different challenges. In large genomes, the challenge is to find the small fraction of non-coding DNA that is conserved, and therefore presumably functional. But as genomes get smaller, and the fraction of functional non-coding DNA increases, the challenge shifts from determining which non-coding sequences are functional to delineating where one regulatory sequence ends and the next begins. Comparative genomic methods have been successfully used to address the first challenge many times, but there are not yet effective methods to address the second challenge.

The key point of this paper is that by going from a small genome like *D. melanogaster* to a bigger genome like *C. capitata* we shift the comparative annotation problem from one we do not yet know how to solve to one that we do. Somewhat counter intuitively, differences in the landscape of non-coding conservation suggest that we can more effectively annotate the function of *D. melanogaster* non-coding DNA by comparing the genomes of two of its distant cousins to each other than by comparing *D. melanogaster* to other *Drosophila* species.

Furthermore, since the basic mechanisms of genome expansion and contraction are not taxon-specific, we think it will be generally true that comparative genomic methods will be more effective in bigger genomes. The added value comes from the “extra” DNA in bigger genomes that consists largely of transposable elements and other families of repetitive DNA that are preferentially found between, rather than within, functional elements.

### Species Selection for Comparative Genomics

It is becoming increasingly common for researchers to sequence multiple species related to a target species of interest in order to assist in its annotation. The general strategy has been to pick multiple species spanning evolutionary distances from the target shown in earlier studies to be ideal for identifying functional elements in the target genome (e.g. [25]). Where possible, smaller genomes are selected to minimize cost.

Our results suggest that rather than avoiding larger genomes, comparative sequencing projects with aspirations to annotate non-coding DNA would benefit from the inclusion of species with large genomes at an optimal evolutionary distance - far enough for non-functional sequences to have significantly diverged, but not so far as to preclude alignment between the reference and comparison genomes. Even where the reference genome is relatively small, CNSs can be identified in the larger genome and mapped back to the reference.

We wish to emphasize that we are not arguing that large genomes are always more useful than small ones or that comparative data is not useful for small genomes. First, the extra DNA in many relatively large genomes is concentrated in heterochromatin and provides limited value for annotation. For example, *Drosophila virilis* has a genome more than twice as large as *D. melanogaster*. However much of the difference in size between the genomes arises from large differences in the amount of DNA in heterochromatic repeat regions [26]. Euchromatic regions of *D. virilis* are modestly bigger than *D. melanogaster* (see Table S2 and Table 2), and have very similar distributions of inter-CNS distances (Figure 5, cyan line).

Although there is much greater separation between regulatory elements in tephritids, some *Drosophila* regulatory elements are flanked by relatively large stretches of non-conserved sequence and can be readily identified by simple comparative methods. For example, comparisons of the *D. melanogaster apterous* locus with its orthologs in three other *Drosophila* species revealed several isolated blocks of sequence conservation, at least one of which corresponds to a transcriptional enhancer [16]. In addition, non-conserved sequences upstream of the *eve* coding gene partition the region into three roughly kilobase sized segments that contain, respectively, the MAS, stripe 3/7 and stripe 2 enhancers. However, this organization is not the norm. Only 13 percent of intergenic regions larger than 2,000 basepairs contain similarly good enhancer candidates (regions of between 500 and 2,000 basepairs flanked by non-conserved regions greater than 300 basepairs – see Table S3).

There may also be patterns in the distribution of conserved elements in the *D. melanogaster* genome that would enable the large-scale direct identification of regulatory sequences. For example, clustering of small conserved blocks has been proposed as a hallmark of regulatory sequences [27], but has not yet been successfully applied on a genome-wide scale.

A major limitation in the development and testing of such methods is the absence of systematic data on regulatory sequence function. Several hundred *D. melanogaster* sequences that drive expression in transgenic reporter assays have been cataloged [28], but the relationship between the borders of these fragments and those of the functional elements they contain is unclear. There is also no catalog of fragments that do not have regulatory function, without which it is impossible to assess the specificity of any prediction method. Ongoing genome-wide experimental screens for enhancers [29], or functional genomic projects such as MOD-ENCODE, may ultimately provide the necessary functional data to enable – or render superfluous - better comparative methods to identify *D. melanogaster* regulatory sequences. In the meantime, we suggest that tephritid genomes provide an effective alternative.

### Using Tephritid Comparisons to Annotate *D. melanogaster*

We are currently sequencing the genomes of *C. capitata* and *B. dorsalis* to extend our pilot study to the entire genome. Despite our success in the *eve* locus, these are not ideal species for comparative annotation of *D. melanogaster*, and there will likely be limits to this endeavor.

Tephritids are more diverged from *Drosophila* than would be ideal. Unfortunately, all surveyed members of the genus *Drosophila* and many species from related genera and families appear to have undergone a substantial genome reduction [5], and there may not be species with large genomes that are more closely related to *Drosophila* than teprhtidis.

At the *Drosophila*-tephritid distance, the identification of orthologous non-coding DNA by standard alignment methods is ineffective. To address this challenge (which, we wish to emphasize, is a product of the suboptimal phylogenetic position of tephritids, not a limitation of the method we are presenting here) we have developed a simple, and surprisingly effective technique for mapping highly diverged tephritid sequences back to the *D. melanogaster* genome. This approach is designed to be broadly applicable to any such mapping of distant homology, regardless of the identity of the best-suited species. However, despite our success with the *eve* locus, it may not be universally possible to map conserved tephritid sequences back to the *D. melanogaster* genome.

Tephritid comparisons will only be effective in identifying *Drosophila* enhancers shared between the families. While the basic similarity of embryonic development between *Drosophila* and tephritids suggests that gene expression patterns during development will be conserved, it is not yet clear how many regulatory sequences are present in both *Drosophila* and tephritids. Furthermore, regulatory sequences that are diverging rapidly [17], and thus are not detectably conserved between the tephritid species we are sequencing, will not be identified in this screen.

### Comparative data may more reliably define enhancer modules than transgenic dissection

The *C. capitata eve* CNSs with activity in *D. melanogaster* generally map to *D. melanogaster* enhancers that drive identical or similar patterns of expression. However, the mapping is not perfect (Figure 8B). In many cases the tephritid CNSs maps to a much larger region of the *D. melanogaster* locus than the corresponding annotated enhancer (MAS, stripe 3/7, stripe 2). In others the CNSs map to portions of an annotated *D. melanogaster* enhancer, with multiple CNSs mapping to the same *D. melanogaster* enhancer (eAPR, CQ).

Some of this is likely due to fuzziness in the CNS mapping. However, we believe the modular organization of the tephritid *eve* locus may also reflect a modular organization of the *D. melanogaster eve* locus that is obscured by the compactness of the *D. melanogaster* genome.

In general, the borders of annotated *D. melanogaster* regulatory elements represent one of many possible sequence fragments with the specified activity. In most cases, a large piece of DNA (∼5 kb) with the desired activity was identified, followed by progressive truncation from both ends until a “minimal element” was defined. However, when the minimal stripe 2 element is deleted from the *D. melanogaster eve* locus, there is still detectable stripe 2 expression [30], suggesting that minimal enhancers do not encompass all of the sequences that contribute to a given expression pattern. While the process of defining minimal enhancers may not keep enhancers intact, we expect that evolution will. Thus we think that conservation is giving us a more reliable guide to the boundaries of functional elements than transgenic assays alone.

The same logic may also apply to enhancers that map to more than one tephritid CNS, such as the *D. melanogaster* eAPR and CQ. While in both cases the two CNSs are close enough that they may represent a single functional enhancer, the ability of both fragments to drive expression in *D. melanogaster* suggests a different modular organization of these enhancers than the reported by the current *D. melanogaster* annotation.

### Conservation of *eve* locus organization

We were struck by the perfect preservation of the relative positions of the *eve* enhancers in *Drosophila*, tephritid, and, based on our previous studies, sepsid genomes [17]. This may simply reflect a relatively low rate of intra-locus inversions and other genomic rearrangements, although there is an inversion of the *eve* stripe 3/7 enhancer in sepsids [17], and the large amount of inter-element sequence in tephritids would seem to foster such locus-scale reorganization. Previous analyses of the stripe 2 and stripe 3 enhancers in transgenes demonstrated that spacing between the elements, but not their ordering relative to the promoter, was required for proper activity [31]. However, the conservation of enhancer order raises the possibility that the ordering of regulatory elements within the *eve* locus is somehow essential for their proper function.

Enhancer interactions may be involved in the regulation of *eve* stripe 7. In *D. melanogaster*, the sequences driving the bulk of *eve* stripe 7 expression overlap those driving stripe 3 (hence the “*eve* stripe 3/7 enhancer”), with proper formation of both stripes achieved by inverse responses to the same pair of repressors at both ends of the embryo [22]. Thus it would seem that stripe 3 and stripe 7 expression should be linked. In addition to the stripe 3/7 element, *D. melanogaster eve* stripe 7 depends on activator sites in the 2 enhancer for full wild-type activity [21]. This, combined with the close physical proximity of the stripe 2 and stripe 3/7 enhancers, has historically rendered the delineation of boundaries between the *D. melanogaster* stripe 2 and stripe 3 enhancers difficult [21], [22]. However, the tephritid stripe 2 enhancer and stripe 3 enhancers are 5 kb apart, with little conserved sequence between them. Curiously, in the tephritid enhancers stripe 7 expression is associated with the stripe 2 enhancer. Perhaps sequences responsible for driving stripe 7 expression are present in both enhancers, and stripe 7 expression is produced by an interaction between enhancers that requires a specific ordering relative to each other and the promoter.

Alternatively, stripe 7 activity in *Drosophila* may represent a lineage-specific reorganization of regulatory information in the compact *eve* locus. The small size of *Drosophila* genomes is believed to be the result of millions of years of genome reduction [32], suggesting that the common ancestor of *Drosophila* and tephritids had a relatively large genome with an organization similar to that of the tephritids. In such a large genome, the modular enhancer model predicts selection against the dispersal of functional transcription factor binding from one enhancer to another. However, the process of genome reduction in the *Drosophila* lineage would have brought previously separated enhancers into close proximity, allowing for the blurring of enhancer boundaries. We suggest that such “enhancer blending” may explain the shift in stripe 7 specification between tephritids and *Drosophila*.

### No fundamental differences between the organization of vertebrate and invertebrate genomes

Although early animal genome sequences documented the extensive similarity of genome content across metazoa, the wide variation in genome sizes – from fly, worm, sea squirt and honey bee with small genomes to human, mouse and rat with big genomes – fostered the impression that vertebrate genomes are big while invertebrate genomes are small, and that these differences in size are accompanied by fundamental differences in genome organization. This notion has been reinforced by the surprising ineffectiveness of comparative genomic methods for identifying regulatory sequences in the invertebrate taxa where they have been applied.

Here we have shown that these impressions are at least in part an artifact of the small size of sequenced invertebrate genomes. While there may be different genome size biases in different taxa [2], [3], there are plenty of invertebrates with big genomes, and these do not look appreciably different – at least with regards to the landscape of non-coding sequence conservation and function – than comparably sized vertebrate genomes.

There may as yet turn out to be fundamental differences in the organization of vertebrate and invertebrate genomes. But we must be careful not to mistake differences in genome size for differences in genome organization. To fully understand the forces that shape genome architecture, it is essential that we explore the diversity of animal genomes as best we can – including the sequencing of large invertebrate genomes.

## Materials and Methods

### Specimens

*Ceratitis capitata*, *Bactrocera cucurbitae* and *Bactrocera dorsalis* stocks were maintained in the Pacific Basin Agricultural Research Station, United States Department of Agriculture, Manoa, Hawaii. *Ragoletis juglandis* adults were live-captured in Tucson, Arizona and then flash-frozen. Samples for genome sizing and genomic DNA isolation were flash-frozen adult flies.

### Genome size determination

Genome sizing methods were adapted from [33]. Two adult heads for each species were dissected into 1.5 mL of Galbraith buffer on ice, homogenized with 15 strokes of an A pestle in a 15 mL Kontes Dounce tissue homogenizer, and filtered through 30 um nylon mesh. Two tephritid heads were combined with 10 *D. melanogaster* heads before homogenization. 7 uL of 1∶10 chicken red blood cells (diluted in PBS) and 50 uL of 1 mg/mL propidium iodide were added and samples were stained for 4 hours rocking at 4 degrees in the dark. Mean fluorescence of co-stained nuclei was quantified on a Beckman-Coulter EPICS XL-MCL flow cytometer with an argon laser (emission at 488 nm/15 mW power). The propidium iodide fluorescence and genome size of *Gallus domesticus* (red blood cell standard, 1,225 Mb) and *D. melanogaster* (174 Mb) were used to calculate the unknown genome sizes.

### Fosmid library preparation

High molecular weight genomic DNA was obtained from approximately 500 mg of frozen adult flies using the Qiagen 500/G Genomic-tip protocol for isolation of genomic DNA from flies (Qiagen Cat. No. 10262). Fosmid libraries were generated according to the Fosmid (40 kb) Library Creation Protocol developed at the DOE Joint Genome Institute (http://www.jgi.doe.gov/sequencing/protocols/prots_production.html) with the following modifications. DNA was end-repaired without hydro shearing, phenol-extracted, and precipitated a second time after gel-purification to increase cloning efficiency. Ligation reactions were incubated overnight at 16°C with T4 DNA ligase then packaged according to the JGI protocol. All libraries are at approximately 5× coverage with an average insert size of 39.5 kb.

### Library screening

Species specific sequence for target genes was obtained by degenerate PCR with primers designed based on *Drosophila* protein sequences, with additional non-*Drosophila* fly sequences used where available. 40 bp overlapping oligonucleotide probes were synthesized by Klenow extension of 24 bp oligos overlapping by 8 bp with radiolabeled dATP/dCTP. Oligos were designed against target gene regions with 50–55% GC and no matches to known PFAM domains. Overgo probes were hybridized in pools of 6–10 probes to high density colony array filters at 60 degrees C overnight as described in [34] and visualized on a Molecular Dynamics Storm 860 phosphorimager. Positive clones were isolated and fosmid DNA was extracted and printed in 12×8 arrays on nylon membranes for hybridization with single overgo probes, protocol as above. 1–3 fosmid clones were shotgun sequenced for each gene in each species, and were selected by EcoRI and BglII restriction mapping from final dot blot positives.

### Sequencing and assembly

Selected fosmids were subcloned and sequenced at the Joint Genome Center; protocols are available at: http://www.jgi.doe.gov/sequencing/protocols/prots_production.html.

Chromatograms were reanalyzed using PHRED v0.020425.c [35], [36] using the phredPhrap Perl script supplied with the CONSED distribution to call bases and assign quality scores. The ARACHNE assembler [37], [38] was then used to build scaffolds (Table S4). After assembly, contigs from fosmids tiling across a given locus for a particular species were further merged by alignment using BLAT [39] (version 25; run with default parameters). Where matches exceeded 98% identity and extended to within 100 basepairs of either: a) both ends of a single contig, or b) one end of both contigs, one of the two sequences for the match region was chosen at random to construct a single representative sequence for the entire region, despite heterozygosity in fosmid libraries.

### Annotation

Protein-coding gene annotation of the fosmids was performed with reference to the Flybase *D. melanogaster* 4.3 annotations. *D. melanogaster* translations were compared to the fosmid sequences translated in six frames using BLASTX. GeneWise [40] was used to construct gene models on scaffolds having hits with e-value≤1e-10, with the query translation as template. Gene models were then filtered by requiring that the model translation find the original *D. melanogaster* query translation among the top hits in a reciprocal BLASTP search against the *D. melanogaster* translation set (e-value threshold 1e-10).

### Determination of endogenous expression patterns in tephritids

Tephritid embryos were collected at the USDA-ARS Pacific Basin Agricultural Research Center. Embryos deposited over either 8 or 24 hours on moist sponges were collected and fixed either immediately, or after aging for 8 or 26 hours as indicated. Fixation was performed as previously described for *D. melanogaster* in 50% fixation buffer (1.3× PBS, 66 mM EGTA pH 8.0) containing 9.25% formaldehyde [21]. 500–1000 bp of coding sequence for each gene were amplified from genomic DNA by degenerate PCR and cloned into the pGEM-T-Easy vector, amplified with M13 forward and reverse primers, and gel-purified with Qia-quick PCR columns. 4 uL of product were used in 20 uL transcription reactions with digoxigenin-11-UTP as described by the manufacturer (Roche DIG RNA Labeling Kit, Cat. No. 11 175 025 910). Probes were then incubated in 100 uL of 1× carbonate buffer (120 mM Na2CO3, pH 10.2) for 20 minutes, and reactions were stopped by addition of 100 uL stop solution (0.2 M NaOAc, pH 6.0). Probes were precipitated with 8 uL of 4 M LiCl and 600 uL EtOH then resuspended in 1 mL hybridization buffer. Hybridizations were performed as described previously with 18–20 hour hybridizations [41]. Embryos were imaged on a Nikon Eclipse 80*i* scope equipped with a Nikon Digital Sight DS-U1 camera.

### Alignment, identification and global analysis of conserved non-coding sequences

Blastz (v7) [42] and TBA/Multiz (v12) [43] were used to compute multiple alignments for entire loci with blastz parameters K: 2200, C: 2, O: 400, E: 30, H: 2000, Y: 3400 and default TBA run parameters. Quantitative assessment of sequence conservation was performed using phastCons with background (non-constrained) rates calculated from each locus alignment separately using phyloFit (HKY85+Gap substitution model), both from the PHASTCONS package [44] (version v0.9.9.6b). Values for the rho parameter from 0.05 to 0.5 were tested, with no appreciable impact on the resulting conservation landscape. Conserved regions were identified by visual inspection of the resulting per-base phastCons posterior probabilities.

Our initial inspection of these loci was based on alignments and analyses of *Drosophila* and vertebrate conservation available through the UCSC genome browser. To eliminate the possibility that the patterns of non-coding conservation might be due to the different parameters used in computing alignments and conservation, we realigned and reanalyzed the *Drosophila* genomes and our tephritid sequence, as well as one percent of the human genome, using a set programs and parameters equivalent to the UCSC alignment and analysis pipeline. No qualitative change in the alignments or resulting estimates of constraint was observed.

### Generation of *D. melanogaster* transgenics

Enhancers were cloned into either the NotI or BglII site in pBΦY-ayeCFP or pBΦY-lacZ vector (modified from pBDP-Gt81, kindly provided by Barret Pfeiffer). Reporter constructs were injected into the *D.melanogaster* attP2 landing pad strain [45] as described [46]. Injection survivors were pooled and red-eyed progeny were screened from the F1 generation.

### Imaging of transgene expression patterns

Transgenic embryos were collected for 4 hours or overnight, as indicated. Fixation, CFP and lacZ probe synthesis, hybridization conditions and microscopy were as described above.

### Mapping tephritid sequences to *D. melanogaster*

Short regions of sequence homology were detected in extremely divergent non-coding comparisons between tephritids and *Drosophila* by windowed sums of BLAST scores and unique K-mer matches as follows. For a given window length from 400 to 2000 base pairs, n×m mappings (where n is the length of the tephritid locus, and m is the length of the *D. melanogaster* locus) were scored as follows. Each window pair was assigned a mapping score as the sum of all pairwise comparisons between the tephritid sequence and each *Drosophila* sequence (*D. melanogaster* in that window and each orthologous sequence region in the 11 other *Drosophila* species) for the following two metrics: A) the scores of all BLAST HSPs above an E-value cutoff of 10, 1 or 0.1 (cutoff of 0.1 reported; bl2seq 2.2.6 from the NCBI blast suite; blastn, all other parameters as default) and B) the number of bases in unique K-mer matches above a cutoff length, as determined by MUMmer (version 3.2, maximal unique matches [-mum] and minimum match length of 8, 10, 12 and 14 tested; 10-mers reported [−l 10]). Results reported are for 600 bp windows.

BLAST scores correspond roughly to the number of matched bases penalized by a function of the number and type of mismatches. Thus, this aggregate summation of uniquely matching K-mers and BLAST HSPs captures and fairly scales both short, ungapped matches of roughly the size of one or a few transcription factor binding sites (K-mers) as well as longer matches potentially representing either conserved or convergent arrangements of multiple short sequences (HSPs). Summation of this aggregate scoring across the up to 12 pairwise comparisons for each tephritid window dampens noise from spurious matches such as those arising from species-specific simple repeat expansions.

Display thresholds for mapping plots were computed as the maximum mapping score of non-coding sequence from the *Drosophila* locus in question compared as described to non-coding sequence from all other tephritid loci. For example, the *eve* cutoff was computed as the highest observed score outside of a coding region for the *Drosophila eve* locus mapping to each of the tephritid *gt, Doc1 and pnr* loci. All above-cutoff mapping window pairs are plotted with opacity scaled to the highest observed score in each locus.

## Supporting Information

Figure S1 Landscape of sequence conservation and inter-family mapping in tephritids and *Drosophilagiant*, *panier* and *dorsocross* loci. Phastcons (version v0.9.9.6b) estimated posterior probabilities of conservation in tephritids (each panel, top) and *Drosophila* (*D. melanogaster* in 12 *Drosophila* alignments, each panel, bottom), as well as aggregate mapping between the two families (see Figure 8; methods). Blue annotations indicate coding regions; orange intervals indicate conserved regions assayed for functionality in this study, interval numbers above are as employed throughout this work; green intervals indicate known *D. melanogaster* enhancers drawn from the Redfly database. A) *B. dorsalis gt* locus (alignment of *B. dorsalis*, *B. cucurbitae*, *R. juglandis*). B) *C. capitata Doc1* locus (alignment of *C. capitata*, *B. cucurbitae*, *R. juglandis*). C) *B. cucurbitae pnr* locus (alignment of *B. cucurbitae*, *C. capitata*, *B. dorsalis*, *R. juglandis*).
(2.87 MB PDF)

Table S1 Coding and non-coding fraction of major animal genomes
(0.03 MB DOC)

Table S2 Genome size and sizes of orthologous region types of *Drosophila* species relative to *D. melanogaster*
(0.04 MB DOC)

Table S3 Intergenic regions (>2,000 bp) with conserved blocks between 500 and 2,000 bp flanked by non-conserved blocks of size = gap size
(0.04 MB DOC)

Table S4 Sequenced Fosmids
(0.05 MB DOC)
