# Supplementary figures and images for: Big Genomes Facilitate the Comparative Identification of Regulatory Elements
Source: PLoS One. 2009 Mar 4;4(3):e4688. doi: 10.1371/journal.pone.0004688 (PMC2650094; doi:10.1371/journal.pone.0004688)

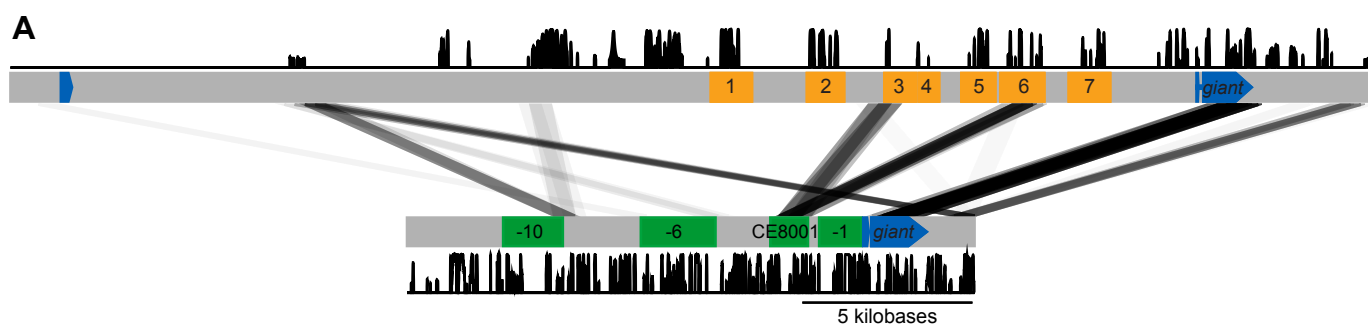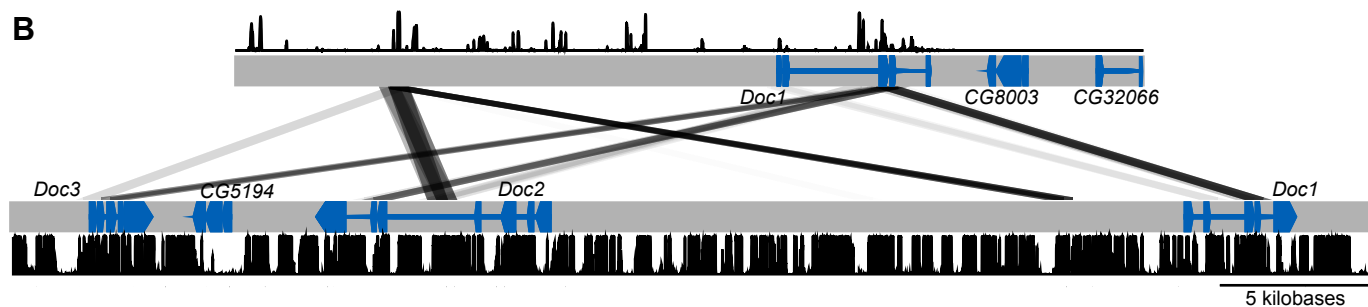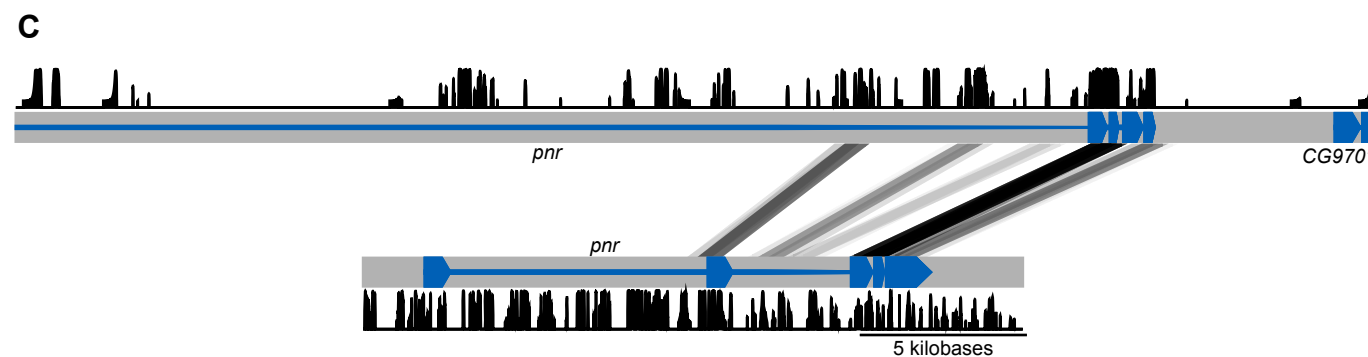

Supplemental Figure 2

Supplement: Figure S1 — Landscape of sequence conservation and inter-family mapping in tephritids and Drosophilagiant, panier and dorsocross loci. Phastcons (version v0.9.9.6b) estimated posterior probabilities of conservation in tephritids (each panel, top) and Drosophila (D. melanogaster in 12 Drosophila alignments, each panel, bottom), as well as aggregate mapping between the two families (see Figure 8; methods). Blue annotations indicate coding regions; orange intervals indicate conserved regions assayed for functionality in this study, interval numbers above are as employed throughout this work; green intervals indicate known D. melanogaster enhancers drawn from the Redfly database. A) B. dorsalis gt locus (alignment of B. dorsalis, B. cucurbitae, R. juglandis). B) C. capitata Doc1 locus (alignment of C. capitata, B. cucurbitae, R. juglandis). C) B. cucurbitae pnr locus (alignment of B. cucurbitae, C. capitata, B. dorsalis, R. juglandis). (2.87 MB PDF) [file pone.0004688.s001.pdf]
