# Supplementary material for: Big Genomes Facilitate the Comparative Identification of Regulatory Elements
Source: PLoS One. 2009 Mar 4;4(3):e4688. doi: 10.1371/journal.pone.0004688 (PMC2650094; doi:10.1371/journal.pone.0004688)
Supplement: Table S1 — Coding and non-coding fraction of major animal genomes (0.03 MB DOC) [file pone.0004688.s002.doc]

Table S1 – Coding and non-coding fraction of major animal genomes

|  | **Coding** | **Non-coding** | **Total** |
| --- | --- | --- | --- |
| ***C. elegans*** | 28,250,000 | 72,000,000 | 100,250,000 |
| ***D. melanogaster*** | 29,500,000 | 139,250,000 | 168,750,000 |
| ***H. sapiens*** | 79,750,000 | 3,028,000,000 | 3,107,750,000 |
