# Supplementary material for: Big Genomes Facilitate the Comparative Identification of Regulatory Elements
Source: PLoS One. 2009 Mar 4;4(3):e4688. doi: 10.1371/journal.pone.0004688 (PMC2650094; doi:10.1371/journal.pone.0004688)
Supplement: Table S2 — Genome size and sizes of orthologous region types of Drosophila species relative to D. melanogaster (0.04 MB DOC) [file pone.0004688.s003.doc]

**Table S2 – Genome size and sizes of orthologous region types of *Drosophila* species relative to *D. melanogaster***

| **Species** | **Genome Size** | **Intergenic** | **Intron** | **Exon** |
| --- | --- | --- | --- | --- |
| *D. simulans* | 1.2 | 1.2 | 1.3 | 0.96 |
| *D. sechellia* | 1.4 | 1.2 | 1.2 | 0.98 |
| *D. yakuba* | 1.4 | 1.3 | 1.1 | 1.00 |
| *D. erecta* | 1.3 | 1.2 | 1.1 | 1.00 |
| *D. ananassae* | 1.9 | 1.2 | 1.4 | 1.00 |
| *D. pseudoobscura* | 1.3 | 1.3 | 1.1 | 1.01 |
| *D. persimilis* | 1.6 | 1.4 | 1.3 | 0.97 |
| *D. willistoni* | 2.0 | 1.7 | 1.7 | 1.01 |
| *D. virilis* | 1.7 | 1.5 | 1.6 | 1.00 |
| *D. mojavensis* | 1.6 | 1.7 | 1.5 | 1.00 |
| *D. grimshawii* | 1.7 | 1.4 | 1.5 | 1.01 |
