# Supplementary material for: Big Genomes Facilitate the Comparative Identification of Regulatory Elements
Source: PLoS One. 2009 Mar 4;4(3):e4688. doi: 10.1371/journal.pone.0004688 (PMC2650094; doi:10.1371/journal.pone.0004688)
Supplement: Table S3 — Intergenic regions (>2,000 bp) with conserved blocks between 500 and 2,000 bp flanked by non-conserved blocks of size = gap size (0.04 MB DOC) [file pone.0004688.s004.doc]

**Table S3 – Intergenic regions (> 2,000 bp) with conserved blocks between 500 and 2,000 bp flanked by non-conserved blocks of size = gap size**

| gap size | number of regions | fraction of total |
| --- | --- | --- |
| 100 | 1450 | 0.13 |
| 200 | 1468 | 0.13 |
| 300 | 1462 | 0.13 |
| 400 | 1421 | 0.13 |
| 500 | 1379 | 0.13 |
| 600 | 1282 | 0.12 |
| 700 | 1139 | 0.1 |
| 800 | 962 | 0.09 |
| 900 | 806 | 0.07 |
| 1000 | 664 | 0.06 |
