# Supplementary material for: Big Genomes Facilitate the Comparative Identification of Regulatory Elements
Source: PLoS One. 2009 Mar 4;4(3):e4688. doi: 10.1371/journal.pone.0004688 (PMC2650094; doi:10.1371/journal.pone.0004688)
Supplement: Table S4 — Sequenced Fosmids (0.05 MB DOC) [file pone.0004688.s005.doc]

**Table S4 – Sequenced Fosmids**

| Gene | Species | ID (Internal ID) | GenBank Accession^1^ |
| --- | --- | --- | --- |
| *eve* | *B. cucurbitae* | Bcuc_evefos1 (FNYC) | FJ710530, FJ710531, FJ710532, FJ710533, FJ710534, FJ710535, FJ710536, FJ710537, FJ710538, FJ710539 |
| *eve* | *B. cucurbitae* | Bcuc_evefos2 (FNYF) | FJ710540, FJ710541, FJ710542 |
| *eve* | *B. dorsalis* | Bdor_evefos1 (FNZH) | FJ710547, FJ710548, FJ710549, FJ710550 |
| *eve* | *C. capitata* | Ccap_evefos1 (FCCS) | FJ710567, FJ710568 |
| *eve* | *C. capitata* | Ccap_evefos2 (FCCT) | FJ710569 |
| *eve* | *R. juglandis* | Rjug_evefos1 (FCUZ) | FJ710579 |
| *eve* | *R. juglandis* | Rjug_evefos2 (FCWF) | FJ710580, FJ710581, FJ710582 |
| *doc* | *B. cucurbitae* | Bcuc_docfos1 (BRUP) | FJ710527, FJ710528, FJ710529 |
| *doc* | *C. capitata* | Ccap_docfos1 (BZYX) | FJ710564, FJ710565, FJ710566 |
| *doc* | *R. juglandis* | Rjug_docfos1 (BZYT) | FJ710574, FJ710575, FJ710576 |
| *doc* | *R. juglandis* | Rjug_docfos2 (FCHG) | FJ710577, FJ710578 |
| *gt* | *B. cucurbitae* | Bcuc_gtfos1 (FNYB) | FJ710543, FJ710544, FJ710545 |
| *gt* | *B. dorsalis* | Bdor_gtfos1 (FCCB) | FJ710598, FJ710551, FJ710552, FJ710553, FJ710554 |
| *gt* | *R. juglandis* | Rjug_gtfos1 (FCCA) | FJ710583, FJ710584, FJ710585, FJ710586, FJ710587 |
| *gt* | *R. juglandis* | Rjug_gtfos2 (FCUW) | FJ710588, FJ710589, FJ710590, FJ710591 |
| *gt* | *R. juglandis* | Rjug_gtfos3 (FCWC) | FJ710592, FJ710593 |
| *pnr* | *B. cucurbitae* | Bcuc_pnrfos1 (BRUF) | FJ710546 |
| *pnr* | *B. dorsalis* | Bdor_pnrfos1 (FCCF) | FJ710555, FJ710556, FJ710557, FJ710558 |
| *pnr* | *B. dorsalis* | Bdor_pnrfos2 (FCGH) | FJ710559, FJ710560 |
| *pnr* | *B. dorsalis* | Bdor_pnrfos3 (FNZF) | FJ710561, FJ710562, FJ710563 |
| *pnr* | *C. capitata* | Ccap_pnrfos1 (BZYY) | FJ710570, FJ710571, FJ710572, FJ710573 |
| *pnr* | *R. juglandis* | Rjug_pnrfos1 (FCUX) | FJ710594, FJ710595, FJ710596 |
